# Ad libitum consumption of milk supplemented with omega 3, 6, and 9 oils from infancy to middle age alters behavioral and oxidative outcomes in male mice

**DOI:** 10.1590/1414-431X2022e12195

**Published:** 2022-10-17

**Authors:** L.B. da Silva, A.J.M. Chaves, M.Q.F.C. Casadevall, O.G.R. de Azevedo, D.S. Macêdo, P.R.L. de Vasconcelos

**Affiliations:** 1 Laboratório de Cirurgia Experimental, Departamento de Ciências Médicas Cirúrgicas, Universidade Federal do Ceará, Fortaleza, CE, Brasil; 2 Núcleo de Pesquisa e Desenvolvimento de Medicamentos, Departamento de Fisiologia e Farmacologia, Laboratório de Neuropsicofarmacologia, Universidade Federal do Ceará, Fortaleza, CE, Brasil; 3 Instituto Nacional de Medicina Translacional, Ribeirão Preto, SP, Brasil

**Keywords:** Aging, Nutraceuticals, Omega 3 fatty acids, Omega 6 fatty acids, Omega 9 fatty acids, Oxidative stress markers

## Abstract

We tested the hypothesis that administration of omega (ω)-9, ω-3, and ω-6 to mice can prevent oxidative alterations responsible for behavioral and cognitive alterations related with aging. Twenty-eight-day-old mice received skim milk (SM group), SM enriched with omega oil mixture (EM group), or water (control group) for 10 and 14 months, equivalent to middle age. Mice were evaluated for behavioral alterations related to depression and memory and oxidative status [brain levels of thiobarbituric acid reactive substances (TBARS), reduced glutathione (GSH), and myeloperoxidase (MPO)]. The 10-month EM group increased immobility time during the forced swimming test compared with control, indicating increased stress response. The 14-month SM- and EM-treated groups increased sucrose consumption compared with control, showing an expanded motivational state. The 14-month SM group decreased the number of rearings compared with the 14-month control and EM groups. The number of entries and time spent in the central square of the open field was higher in the 10-month EM group than in the control, revealing an anxiolytic-like behavior. TBARS decreased in the hippocampus and striatum of the 10-month EM group compared with the control. A similar decrease was observed in the striatum of the 10-month SM group. GSH levels were higher in all 14-month treated groups compared with 10-month groups. MPO activity was higher in the 14-month EM group compared with the 14-month control and SM groups, revealing a possible pro-inflammatory status. In conclusion, omega oils induced conflicting alterations in middle-aged mice, contributing to enhanced behavior and anxiolytic and expanded motivational state, but also to increased stress response and pro-inflammatory alterations.

## Introduction

In recent years, significant advancements in life expectancy have been made. Neurodegenerative diseases pose relevant challenges to increased life expectancy, but there are no effective preventive interventions. It is generally accepted that the aging-related oxidative-inflammatory process could be the harmful consequence of the high reactivity of free radicals produced in cells/tissues by oxygen metabolism (1). Indeed, the brain's chronic low-grade inflammation (such as that related to chronic microglial activation) contributes to the pathophysiology of neurodegenerative diseases, such as Alzheimer's and Parkinson's diseases and multiple sclerosis (2). The hippocampus and striatum are the brain areas most commonly related to neurodegenerative diseases (3).

Eicosapentaenoic (EPA) and docosahexaenoic (DHA) fatty acids are the main omega 3 (ω-3) polyunsaturated fatty acids (PUFAs) and are found in fish and certain plants. These PUFAs play an important role in the central nervous system (CNS) and are necessary to the normal development of this system. Therefore, their deficiency can harm brain functions and development (4). Thus, adequate dietary intake of ω-3 PUFAs may prevent cognitive decline and mitigate aging-related physiological disorders, including neurodegenerative diseases such as Alzheimer's and Parkinson's (5,6).

Regarding ω-6 PUFAs, arachidonic acid (AA) mediates pro-inflammatory status through eicosanoid biosynthesis by cyclooxygenase enzymes (COX-1 and COX-2). The AA derivatives include leukotrienes, thromboxane, and prostaglandins: molecules that lead to inflammation, free radicals production, vasoconstriction, platelet aggregation, and neurological deterioration (7). In contrast, some AA derivatives, such as lipoxins, play a beneficial role in the nervous system. Specifically, lipoxin A4 (LXA4) reduces brain damage after traumatic brain injury and decreases the release of pro-inflammatory cytokines, including tumor necrosis factor (TNF)-α, interleukin (IL)-1β, and IL-6 (8).

ω-9 monounsaturated fatty acids (MUFAs) protect against lipid peroxidation. The cell membranes that are rich in MUFAs are less susceptible to peroxidation by free radicals than those rich in PUFAs, possibly due to the smaller number of double bonds present in MUFAs (9). Furthermore, they also act in the synthesis of the myelin basic protein (MBP) (10), suggesting a central role for this fatty acid in neuronal function.

In a recent study, ω-3 PUFA's prevented hippocampal neuronal cells from increased inducible nitric oxide synthase (iNOS), nuclear factor kappa B (NF-kB) (p50/p65), IL-6, and nitrite induced by the viral mimetic polyinosinic:polycytidylic acid (poly I:C) challenge, highlighting the involvement of canonical NF-kB pathway inhibition in ω-3 PUFA's effects (11). Furthermore, as mentioned above, ω-6 PUFAs and ω-9 MUFAs may protect from inflammatory and oxidative alterations. Since NF-kBp65 is a transcription factor for inflammatory and oxidative alterations, we hypothesized that the administration of ω-9, ω-3, and ω-6 to healthy/control mice can prevent this early inflammation and oxidative alterations that contribute to the onset of neurodegenerative diseases related to the aging process. To this end, our primary outcome was the behavioral and cognitive alterations associated with long term administration of a mixture of canola, fish, and sunflower oils, which provide omega 3, 6, and 9 oils. Our secondary outcome was the oxidative activity, e.g., lipid peroxidation, reduced glutathione (GSH), and inflammatory activity, e.g., myeloperoxidase activity in the hippocampus and striatum of these mice.

## Material and Methods

### Animals

Seventy-two male Swiss mice *(Mus musculus*) from the Central Animal Facility maintained at the Experimental Surgery Laboratory (LABCEX) of the Universidade Federal do Ceará (UFC) were used. The mice (aged 21 days old; mean weight of 10 g) were randomly housed 6 per cage (standard polycarbonate, 42×20.5×20 cm) and kept at ambient conditions (23±1°C, humidity 60±5%) with 12/12-h day/night cycles. The UFC ethics committee approved the research protocol (63/2013), which was conducted following the NIH Guide for the Care and Use of Laboratory Animals and the Brazilian College of Animal Experimentation (COBEA).

### Nutraceutical preparation

We used a mixture of canola, fish, and sunflower oils, which provide omega 3, 6, and 9 oils, in the proportions ω-6:ω-3 (1.4:1.0) and ω-9:ω-6 (3.7:1.0). The oil mix was added to lactose-free skim milk of the Ultra High Temperature (UHT) type at a concentration of 3%. We used this fatty acid mixture based on previous anti-inflammatory effects (12).

### Experimental design

After weaning on postnatal day 21, the 72 mice were equally and randomly distributed in 12 cages containing filtered water and standard chow (CR1-NUVILAB Nuvital^®^, Brazil) *ad libitum*. After an adaptation period of 7 days, the cages were divided and the mice were placed into three groups: skim milk without lactose (SM group), skim milk enriched with omega oil mixture (EM group), or water (control group). The animals were treated for 10 and 14 months, corresponding to the beginning and end of middle age in mice, respectively. We registered the animals' daily liquid intake in each cage by subtracting the initial volume of liquid present in each bottle (300 mL) by the final volume registered at the end of each 24-h period. This volume was registered every day at 10:00 am. To obtain the daily liquid intake, the volume difference (initial minus final) was divided by the number of animals in each cage, i.e., 6 mice. We also registered the animal's weight every 30 days until the end of the experimental protocol.

The milks were processed by the “Companhia Brasileira de Laticínios” (Brazil) and packed in 200-mL Tetra Pak^®^ packages. All liquids were offered in drinking bottles *ad libitum.* All groups continued to receive standard solid feed *ad libitum*. In the SM and EM groups, drinking water was substituted with skim and enriched milk during the entire treatment. Since our protocol was based on a long-term administration of the solutions, the *ad libitum* administration was chosen to reduce the stress associated with the gavage procedure.

**Figure 1:**
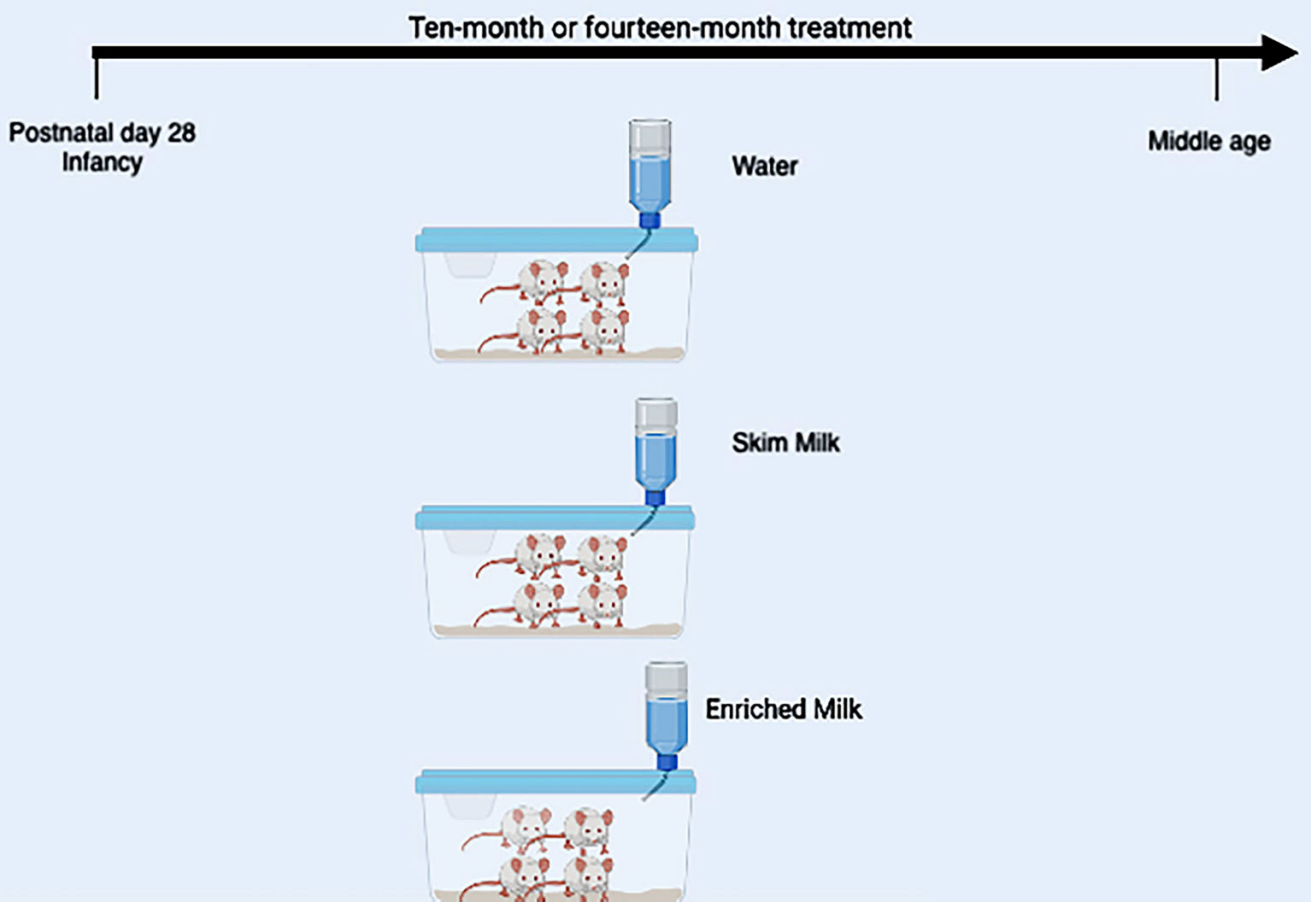
Timeline of the experimental design of the study.

Every 12 h, all bottles were changed. First, the milk boxes were opened, and all their contents were immediately dispensed in sterile drinking bottles. Following the 12-h cycle, residual liquids were discarded, and the drinking bottles were sent for autoclaving. After 10 and 14 months from the start of the treatment, we selected half of the animals from each group/time of treatment to perform the behavioral tests. Figure 1 presents a summary of the study's experimental design.

### Behavioral tests

#### 2.4.1 Y-maze

This test was used to assess spatial working memory, that is, exploratory cognitive behavior, through the spontaneous alternation performance. The apparatus consisted of an acrylic labyrinth, built in a Y shape, with three identical arms 45.0 cm long, 35.0 cm high, and 10 cm wide, each converging at an equal angle of 120°. Each mouse was placed at the end of an arm and allowed to explore the environment for 8 min. First, we registered the arms' sequence entrance and analyzed it to determine the number of entries in the arm without repetition, considered correct alternations. An alternation was deemed correct if the animal visited a new arm and did not return to the previously visited arm. The maximum number of alternations was the total number of entries in the arms minus two (n-2), and the percentage of alternations was calculated as: (number of correct alternations / maximum number of alternations) × 100.

#### Forced swimming

For this test, we placed the animals individually in an acrylic cylinder (50 cm high, 18 cm in diameter) containing 30 centimeters of water depth at room temperature (25±1°C). After a habituation period of 1 min, the animals' immobility time, in seconds, was counted for 5 min of testing. An increase in immobility duration indicates depression-like behavior or depression associated with negative schizophrenia-like symptoms.

#### Sucrose preference

This test assesses the ability to feel pleasure and motivation. Seventy-two hours before the test, the animals received a 1% (w/v) sucrose solution through two water dispensers. Twenty-four hours later, one of the bottles was replaced by a drinking fountain with filtered water. After 24 h, mice were deprived of food and water for further 24 h. After this period, the animals were housed individually in cages containing two drinking fountains: one with 100 mL of sucrose solution and the other with 100 mL of filtered water. One hour later, we registered the solutions' final volumes, and the following formula was used for sucrose consumption: Sucrose preference = (sucrose consumption) × 100% / (sucrose consumption + water consumption).

#### Open field test

The animal's locomotion and exploratory activity were evaluated using a square-shaped acrylic apparatus (30×30 cm, 15-cm high walls), divided into 9 squares with equal areas. This test also allows for measuring the animals' emotionality and anxiolytic behavior. The animals were placed in the center of the field, where they were left for 1 min for exploring (habituation period), followed by another 5 min (test period). The following parameters were observed: number of entries into the central zone, total time spent in the central zone (anxiolytic behavior), number of crossings between lines (horizontal exploratory activity), and number and time of rearings (vertical exploratory activity). A 20% ethanol solution was used to clean the device after each animal.

### Neurochemical tests

#### Brain areas

The animals were rapidly killed by decapitation in a guillotine, which was carried out professionally and compassionately by qualified and experienced staff, following the international guides for the care and use of laboratory animals. For euthanasia, a physical method was preferred over chemical agents because of the potential impact of sedatives or anesthetics on synaptic mechanisms. After the sacrifice, the brain was immediately removed and dissected in a Petri dish lined with aluminum foil and kept on ice. Hippocampi and striatum were dissected and stored at -70°C in identified microtubes until use. First, the brain was placed with the ventral side facing the metal plate for hippocampi dissection. Then, small, curved forceps were placed between the cerebral halves in a closed position and gently opened. With this procedure, we can find the initial, white-colored part, the corpus callosum, and the hippocampus. Hence, we can see the hippocampus after the gentle removal of the cortex. Next, we opened the brain in coronal sections for striatal tissue, comprising caudate, putamen, and accumbens.

#### Lipid peroxidation

Lipid peroxidation was assessed by measuring thiobarbituric acid reactive substances (TBARS) in homogenates. To this end, the malondialdehyde (MDA) content was calculated in the tissue. The samples were homogenized with monobasic potassium phosphate buffer (pH=7.4). First, 63 μL of the homogenate was mixed with 100 μL of 35% perchloric acid and centrifuged at 5478 *g* for 15 min at 4°C. Then, 150 μL of the supernatant was removed, mixed with 50 μL of 1.2% thiobarbituric acid, and heated in a boiling water bath for 30 min. After cooling, lipid peroxidation was determined by absorbance at 535 nm and is reported as µmol of malonaldehyde (MDA)/mg protein.

#### Reduced glutathione levels (GSH)

GSH levels were assessed to estimate endogenous defenses against oxidative stress. The method is based on Ellman's reagent (DTNB) reaction with free thiol groups. The brain samples were diluted in 0.02 M EDTA buffer (10% w/v) and added to a 50% trichloroacetic acid solution. The homogenate supernatant was collected after centrifugation (1006 *g*, 15 min, 4°C). The samples were mixed with 0.4 M of a tris-HCl buffer, pH=8.9, and 0.01 M of DTNB. GSH levels were determined by spectrophotometry at 412 nm, the calculation of which was based on a standard glutathione curve and reported as ng GSH/mg protein.

#### Myeloperoxidase activity (MPO)

Myeloperoxidase is a highly oxidative enzyme. The extracellular activity of this enzyme provides an estimate of oxidative stress in inflammatory conditions. For this analysis, the homogenates were made using a 0.5% solution of hexadecyltrimethylammonium bromide (HTAB) in 50 mM phosphate buffer pH=6.0 (1 mL/50 mg of tissue) and centrifuged (1789 *g*, 15 min, 4°C). Phosphate buffer (50 mM, pH=6,0) containing 0.167 mg/mL of 9-dianisidine dihydrochloride and 0.0005% hydrogen peroxide was added to 30 µL of the supernatant. The absorbance was determined at 450 nm in two stages, 0 and 3 min, to estimate the MPO activity (U MPO·min^-1^·mg of tissue^-1^).

### Statistical analysis

GraphPad 9.0.1 software (USA) was used to analyze the data and create the graphs. First, the Kolmogorov-Smirnov test verified the normal distribution of data. Next, sample size calculation was performed using Resource Equation Approach for two-way ANOVA. Then, we performed two-way ANOVA followed by the Tukey *post hoc* test considering as factors “time of administration” (10 and 14 months) and “milk supplementation” (water, skim milk, and enriched milk). The alpha level was set at 0.05.

## Results

**Figure 2:**
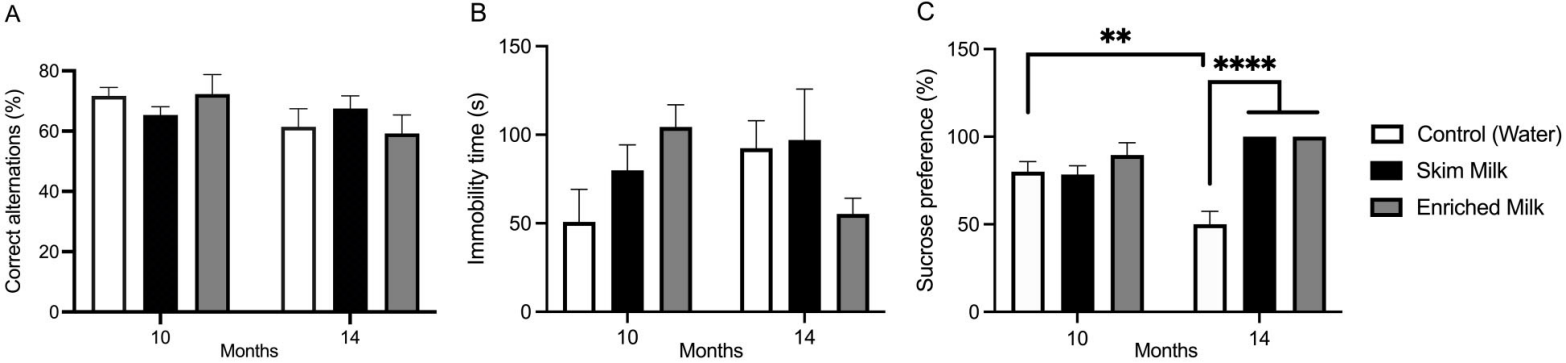
Alternations in the Y maze (**A**), immobility time in forced swimming (**B**), and sucrose preference (**C**). Data are reported as means±SE of 6-12 animals/group. Data were analyzed by two-way ANOVA considering the factors “time of administration” (10 and 14 months) and “milk supplementation” (water, skim milk, and enriched milk). **P<0.01, ****P<0.0001.

The animals treated with EM for 10 or 14 months presented no alterations in % spontaneous alternations (Figure 2A), a parameter used to evaluate spatial working memory (two-way ANOVA interaction: [F (2, 45)=1.540, P=0.2254]). Immobility time in the forced swimming test (Figure 2B), a measure of depressive-like behavior, increased by 2-fold (not significantly) in mice treated with EM for 10 months compared to the control group. On the contrary, mice treated for 14 months with EM had a non-significant 1.7-fold decrease in immobility time in relation to the SM and the control groups (two-way ANOVA: [F (2, 34)=3.840, P=0.0327]). Sucrose preference (Figure 2C) was higher in mice treated for 14 months with EM and SM compared to control (P<0.0001). We also observed that 14-month control mice presented a significant decrease (P<0.01) in sucrose preference compared with the 10-month control group (two-way ANOVA interaction: [F (2, 36)=11.87, P=0.0001], main effect of “milk supplementation” [F (2, 36)=16.12, P<0.0001]).

**Figure 3:**
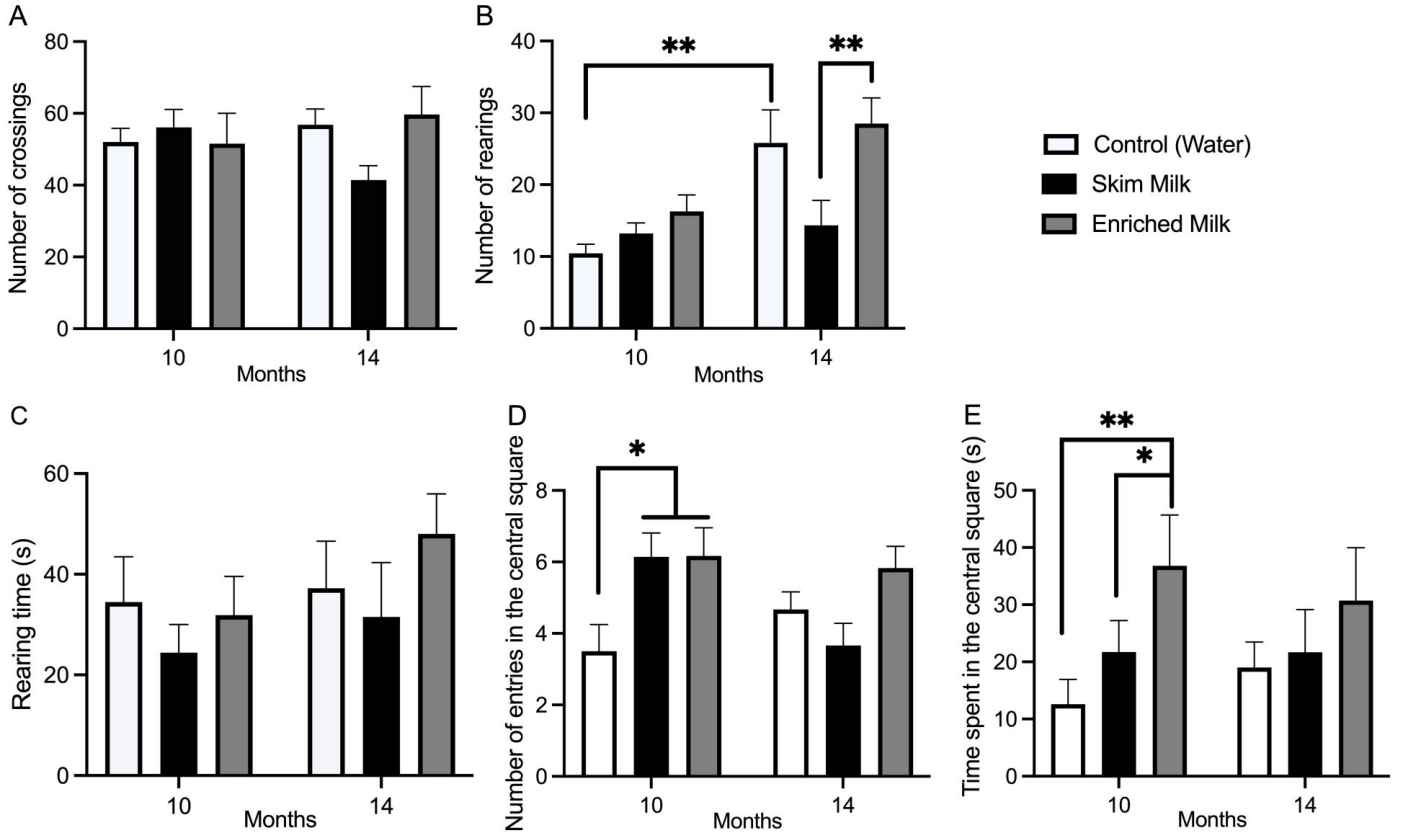
Representation of behavioral parameters obtained in the open field test. Number of crossings (**A**) and number of rearings (**B**) during 5 min, rearing time (**C**), and number of entries (**D**) and time spent (**E**) in the central square of the field. Each bar represents means±SE of 6-12 animals/group. Data were analyzed by two-way ANOVA considering the factors “time of administration” (10 and 14 months) and “milk supplementation” (water, skim milk, and enriched milk). *P<0.05, **P<0.01.

The analysis of locomotor activity revealed no alterations in the number of crossings (Figure 3A) in the open field test (two-way ANOVA interaction: [F (2, 36)=2.060, P=0.1426]). On the other hand, the number of rearings (Figure 3B) significantly increased in mice treated with EM for 14 months compared to SM (P=0.0111). Furthermore, we observed that 14-month control mice had an increased number of rearings compared with 10-month control (P=0.0033) (two-way interaction: [F (2, 36)=4.176, P=0.0234]). Rearing time (Figure 3C) did not vary between groups in any of the treatment protocols (two-way ANOVA: [F (2, 36)=0.3061, P=0.7381]). The evaluation of anxiolytic-like behavior by the analysis of the number of entries (Figure 3D) and time spent (Figure 3E) in the central square of the open field showed an increased number of entries in 10-month EM- and SM-treated mice compared to control (P<0.05). In contrast, the time spent in the central square was higher in mice treated for 10 months with EM compared to SM (P<0.05) and control (P<0.01).

**Figure 4:**
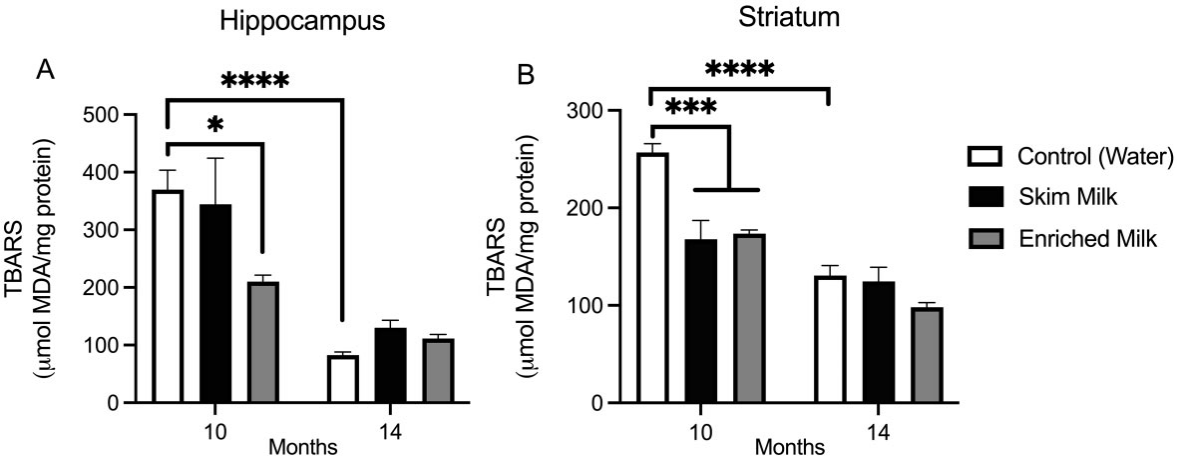
Lipid peroxidation in the hippocampus (**A**) and striatum (**B**). Each bar represents means±SE of 6-12 animals/group. Data were analyzed by two-way ANOVA considering as factors “time of administration” (10 and 14 months) and “milk supplementation” (water, skim milk, and enriched milk). *P<0.05, ***P<0.001, ****P<0.0001.

The evaluation of oxidative alterations in the hippocampus (Figure 4A) revealed decreased lipid peroxidation in mice supplemented for 10 months with EM compared with control (P=0.0115). We also observed decreased lipid peroxidation in the hippocampus of 14-month control mice compared with 10-month control (P<0.0001) (two-way ANOVA interaction: [F (2, 31)=5.555, P=0.0087], main effect of “time of administration” [F (1, 31)=68.20, P<0.0001]). In the striatum (Figure 4B), we detected decreased lipid peroxidation in mice treated for 10 months with EM and SM compared to control (P<0.001). Similar to what was observed in the hippocampus, we also saw lower lipid peroxidation in the striatum of 14-month control mice compared to 10-month controls (two-way ANOVA interaction: [F (2, 31)=5.954, P=0.0070], main effect of “time of administration [F (1, 31)=64.28, P<0.0001], and “milk supplementation” [F (2, 31)=12.74, P=0.0001]).

**Figure 5:**
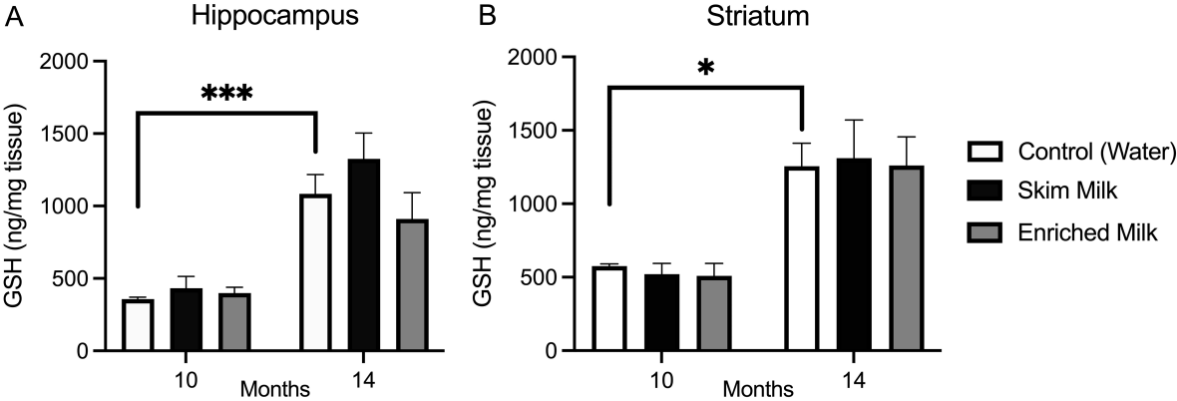
GSH levels in the hippocampus (**A**) and striatum (**B**). Each bar represents means±SE of 6-12 animals/group. Data were analyzed by two-way ANOVA considering as factors “time of administration” (10 and 14 months) and “milk supplementation” (water, skim milk, and enriched milk). *P<0.05 and ***P<0.001.

Regarding GSH levels, we observed higher hippocampal (Figure 5A) and striatal (Figure 5B) levels of this antioxidant in control mice treated for 14 months compared with mice treated for 10 months (hippocampus: 10-month control *vs* 14-month control, P=0.0010; striatum: 10-month control *vs* 14-month control, P=0.0344).

**Figure 6:**
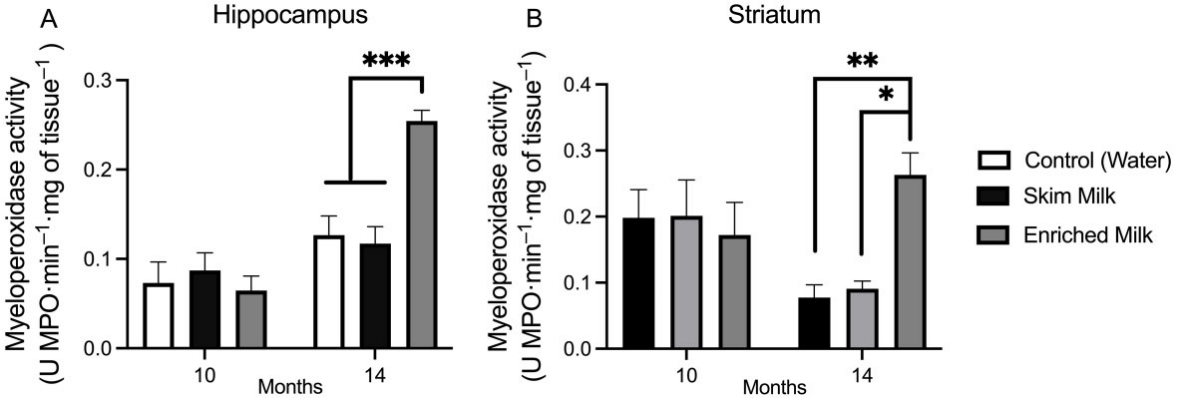
Myeloperoxidase activity in the hippocampus (**A**) and striatum (**B**). Each bar represents means±SE of 6-12 animals/group. Data were analyzed by two-way ANOVA considering as factors “time of administration” (10 and 14 months) and “milk supplementation” (water, skim milk, and enriched milk). *P<0.05, **P<0.01, and ***P<0.001.

Hippocampal MPO activity (Figure 6A) was significantly higher in the 14-month EM-treated group compared with the control and SM-treated groups (P<0.001). Similarly, striatal MPO activity (Figure 6B) significantly increased in the 14-month EM-treated group compared to the control (P<0.01) and SM-treated groups (P<0.05).

**Figure 7:**
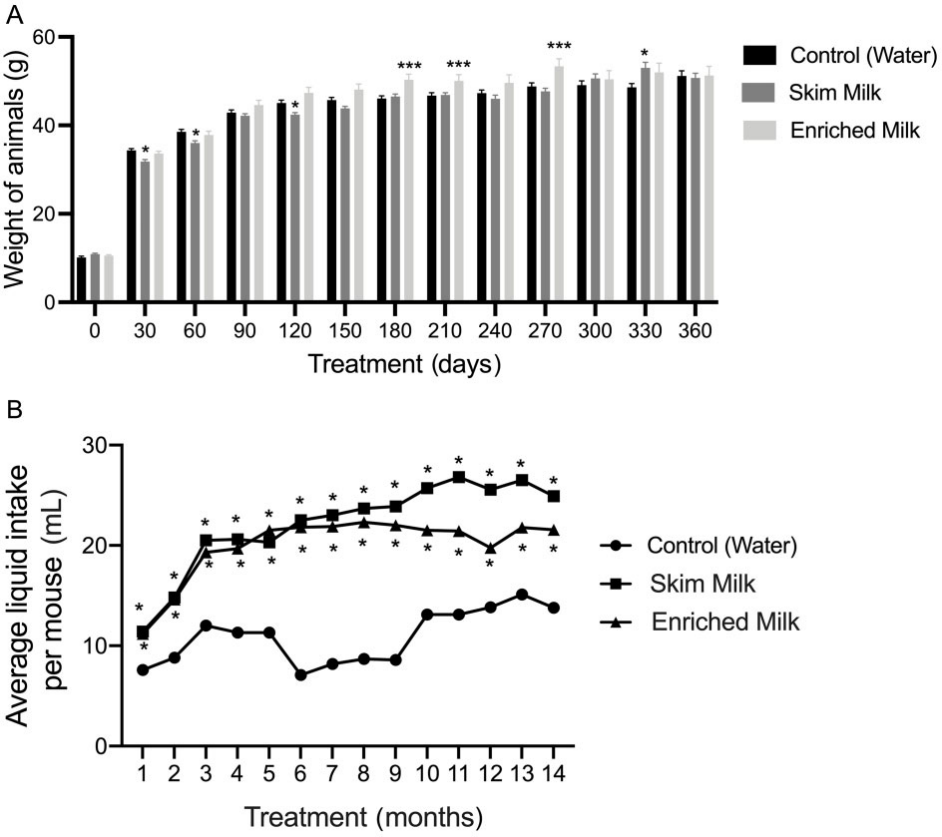
Weight of mice during treatment protocol (**A**) and average liquid intake per mouse (**B**). Each bar represents means±SE of 6-12 animals/group. Data were analyzed by two-way ANOVA. *P<0.05, ***P<0.001 compared with the control group in each day of treatment.

The evaluation of weight (Figure 7A) revealed that SM-treated mice had a significant weight decrease on days 30, 60, and 120 after treatment onset compared to control (P<0.05). On the other hand, on days 180, 210, and 270, animals treated with EM presented a significant weight gain compared to control (P<0.001). On day 330, the SM-treated group presented a significant weight gain compared with controls (P<0.05) (two-way ANOVA interaction: “time of administration” *vs* “treatment groups” [F (24, 719)=2.422, P=0.0002]). In the analysis of daily liquid intake (Figure 7B), we detected significantly increased average liquid intake in the animals treated with SM and EM compared to the control in each month of evaluation (P<0.05). The mean daily liquid consumption per mouse of the control throughout the months was 10.9 mL, while the means for groups SM and EM were, respectively, 22.2 and 20 mL (two-way interaction: “time of administration” *vs* “treatment groups” [F (24, 719)=2.422, P=0.0002]).

## Discussion

Our results revealed that the 10-month EM group increased stress response while presenting an anxiolytic-like behavior. These animals also presented decreased lipid peroxidation. On the contrary, the 14-month EM group presented a pro-inflammatory status revealed by increased MPO activity. These results let us partially reject our hypothesis and conclude that prolonged administration of omega oils to control disease in middle-aged mice seems to cause conflicting alterations, contributing to enhanced behavioral performance and anxiolytic-like and expanded motivational state and increased stress response and pro-inflammatory alterations. Probably, the use of mice at control conditions may have contributed to this partially negative effect of omega oils, revealing the need for studies addressing the necessity for the supplementation of these oils to be done under controlled conditions.

Aging decreases cognitive performance, even without a disease condition (13). The most frequent changes include memory and executive functions, making it more challenging to perform new learning tasks and slow information processing (14). Therefore, the present study was designed to assess the impact of aging on the behavioral and cognitive performance of mice, as well as the possible nootropic effect of the EM by the evaluation of cognitive, motivational, anxiolytic, and locomotor alterations followed by the quantification of hippocampal and striatal oxidative/inflammatory markers.

Our results revealed that the long-term administration of SM and EM, from infancy to middle age, caused no working memory alterations in the Y-maze test in animals under control conditions. However, previous studies have demonstrated the beneficial effect of ω-3 in relation to memory and learning (13). Ferraz et al. (13) reported that supplementation with fish oil (3.0 g/kg of body weight, with lipid emulsion containing 12% EPA and 18% DHA) from the early stages of development prevented the occurrence of memory and learning deficits in animals subjected to a model of restraint stress. Also, supplementation with ω-3 for 2 months improved working memory, assessed by the Y-maze test, in treated elderly animals (15).

Loef and Walach (16) conducted a systematic review of studies that reported a positive association between the ω-6/ω-3 ratio and dementia or cognitive decline. In 13 studies, the ω-6/ω-3 dietary ratio was shown to affect brain composition, the clinical course of Alzheimer's disease, and mouse behavior. Evidence from 14 clinical studies supports an association between the high ω-6/ω-3 ratio, cognitive decline, and incidence of dementia. The dietary supplementation with DHA associated with a low ω-6/ω-3 ratio decreased the brain ω-6/ω-3 ratio and lowered the brain levels of amyloid beta-peptide and Tau protein. Thus, it is suggested that a low ω-6/ω-3 diet ratio may have critical effects on maintaining cognitive function and preventing dementia (17). The present findings can perhaps be explained by the fact that the animals were not subjected to environmental challenges, such as stress or diet deprivation of fatty acids. Therefore, there was probably no deficiency of fatty acids in our experimental groups. Hence, we observed no additional benefit in working memory with the supplementation of omega oils.

Depression is the most prevalent and treatable mental health problem in the elderly. In addition, apathy or a loss of motivation are signs of depression linked to various negative effects, including visible cognitive impairment, diminished everyday activity, and a lack of awareness of one's own physical and cognitive impairment (18). In the present study, we observed that the 14-month control mice presented a 1.8-fold increase in immobility time in the forced swimming test in relation to the 10-month control, indicative of a putative depressive-like behavior related to aging. Also, the 14-month EM group presented a 1.7-fold decrease in immobility time, despite being non-significant, compared to control. Surprisingly, mice from the 10-month EM group had higher immobility, despite being non-significant, compared to the 10-month control. In a recent study on the preventive effects of ω-3 PUFAs against schizophrenia-like alterations induced by the neonatal exposure to the viral mimetic polyinosinic:polycytidylic (Poly I:C), the authors found that adolescent male mice treated with ω-3 PUFAs and not exposed to the neurodevelopmental hit with poly I:C (i.e., at control conditions), presented impaired working memory and social preference. These authors concluded that under control conditions, ω-3 administration might induce behavioral deficits, whose mechanisms must be explored in future studies (19).

On the other hand, corroborating the results of reduced immobility observed in the 14-month EM group, Lakhwani et al. (20) evaluated the effect of ω-3 alone and compared it with standard antidepressant therapy fluoxetine, showing that the chronic administration of ω-3 significantly affects immobility and swimming behavior compared to control. The response of the combination of ω-3 PUFAs and fluoxetine was substantially greater than fluoxetine alone in altering the behavioral activity of rats in this test. These findings indicated that ω-3 PUFAs have antidepressant properties independently. Huang et al. (21) also showed that rats fed a diet rich in ω-3 for six weeks, compared to animals fed standard chow, had shorter immobility times and increased swimming time and climbing during the forced swimming test. The diverging results in immobility time observed here concerning the treatment effects with ω-3 PUFAs for 10 months and 14 months must be better evaluated in future studies.

As previously mentioned, lack of motivation and anhedonia are behavioral alterations observed in depression and are useful in predicting an antidepressant response (22). The sucrose preference test is widely used for the preclinical evaluation of anhedonia (23). We found that the 14-month control group decreased sucrose consumption compared to the 10-month control, revealing aging-induced anhedonia. The decreased sucrose preference and increased immobility in the forced swimming test in the 14-month control compared to the 10-month control confirmed a depressive-like phenotype in 14-month control mice. Both SM and EM prevented reduced sucrose preference in 14-month mice. Therefore, we can hypothesize that ω-3, ω-6, and ω-9 supplementation protects against the early onset of depressive-like behavior associated with aging since only the EM group presented decreased immobility time and increased sucrose preference.

The lipid mixture used in the present study had a low ω-6/ω-3 ratio, providing an anti-inflammatory characteristic to the mixture, and a high ω-9/ω-6 ratio, offering an antioxidant effect. Therefore, this mixture could decrease low-grade chronic inflammation and consequently improve depressive symptoms. Husted and Bouzinova (24) showed the putative correlation between high ω-6/ω-3 ratio, low-grade inflammation, and depression. Indeed, these authors revealed that high ω-6/ω-3 ratio induced pro-inflammatory cytokines related to depressive symptoms. Furthermore, high ω-6/ω-3 ratio also activated the hypothalamic-pituitary-adrenal axis and reduced serotonin precursors' availability, corroborating the findings of inflammation as a key player in depression onset.

Several morphophysiological changes that occur during the aging process are related to the decreased functional capacity of the elderly (25). Here, the open field test was carried out to analyze the effect of aging and supplementation of milk enriched with omega oils on the animal's locomotor and exploratory activity. We observed no alterations in horizontal locomotor activity (number of crossings), which agrees with previous studies (26).

Regarding the assessment of vertical exploratory behavior, our results revealed that the EM group had a better performance in rearing behavior compared to animals that received only SM. Bäckman et al. (27) showed that rearing behavior is associated with motivation and excitability for exploration, which is impaired with aging. Additionally, we observed that the EM group treated for 10 months presented increased time spent in the open field center, suggesting anxiolytic-like behavior. Indeed, studies on exploratory behavior are relevant for measuring the animals' emotionality. The locomotor activity associated with emotionality can be assessed by observing the number of crossings in the center and the time in the center in the open field test.

We also assessed the effect of SM and EM on oxidative parameters in the hippocampus and striatum. Both brain areas are relevant for neurodegenerative disorders since the hippocampus is a critical brain region for learning and memory and because altered neurogenesis in the adult hippocampus is an important early event in Alzheimer's disease (28). Furthermore, evidence indicates striatal impairments (caudate, putamen, and nucleus accumbens) in neurodegenerative conditions (29).

Excessive formation of brain reactive oxygen species (ROS) can lead to mitochondrial protein oxidation, DNA and RNA oxidation, neuronal dysfunction, and cell death (30). Several antioxidant systems can restore oxidative homeostasis, including enzymatic superoxide dismutase and catalase, GSH, ascorbate, and α-tocopherol. However, in some cases, the high production of ROS and high lipid peroxidation levels may exceed the power of the cell's antioxidant defenses, resulting in oxidative imbalance (31,32).

Due to its bio-functional potential, bioactive milk peptides are widely studied for their CNS benefits (32). Hence, several milk peptides have antioxidant activity. For example, Zemel et al. (33) showed that individuals with metabolic syndrome who had a diet high in dairy products showed decreased oxidative stress markers. Furthermore, in healthy overweight and obese adults, dairy-based shakes significantly reduced circulating markers of oxidative stress and inflammation (TNF-α, IL-6, monocyte chemotactic protein 1, and C-reactive protein) and raised levels of adiponectin.

In the present study, we observed that 10-month EM-treated mice had lower hippocampal lipid peroxidation, whereas in the striatum, this alteration was observed in both SM and EM groups. This effect of SM in the striatum may reflect the presence of bioactive milk peptides. Besides, the effects of the EM group are probably related to the supplement of ω-3 and ω-9, which protect against lipid peroxidation.

Aging is associated with increased ROS production. On the other hand, this study has demonstrated lower levels of TBARS in 14-month treated groups compared with 10-month groups. The higher levels of GSH can explain this finding in the hippocampus and striatum observed in all groups of 14-month treated mice. However, during the aging process, one would expect a decrease in cellular antioxidants, such as GSH, and not the increase observed in the control group.

McIntosh et al. (34) demonstrated that milk proteins protect against intestinal tumors in male rats. The protective effect of these proteins was related to higher intracellular concentrations of GSH in hepatocytes in rats fed with whey and casein. Ebaid et al. (35) showed that supplementation with whey protein (100 mg/kg for 15 days pre- and post-wound) significantly reduced wound healing time in diabetic rats, accompanied by a reduction in oxidative stress, as they observed decreased levels of MDA, ROS, and nitric oxide and increased levels of GSH in the injured tissue. Therefore, in agreement with previous studies, we observed higher levels of GSH in the hippocampus and striatum of 14-month SM- and EM-treated groups.

As previously mentioned, MPO is a component of the inflammatory response, and the uncontrolled formation of these compounds leads to protein and lipid oxidation. MPO activity increases during aging and during recruitment of inflammatory cells (36). Son et al. (36) reported an increase in MPO with aging. These authors showed that both the activity and the MPO protein level were increased in rats fed *ad libitum* food during aging. On the other hand, a smaller amount or lack of MPO can also cause an increase in oxygen metabolism and, therefore, a higher-than-normal production of ROS, leading to a greater susceptibility to infection and increased risk of cancer in humans (37).

This study showed increased MPO activity in the hippocampus and striatum of the 14-month EM group compared with the SM group and control. This result was surprising since we did not observe lipid peroxidation and detected increased GSH levels in mice from the 14-month EM group.

A hypothesis for the increased MPO activity in the 14-month EM group could be the influence of these oils in maintaining the biological role of neutrophils during aging associated with neutrophil recruitment caused by ω-9. On the other hand, we speculate that the antioxidant properties of these lipids prevented ROS formation from the activation of neutrophils. Based on the involvement of MPO in oxidative/inflammatory alterations, the meaning of the increased brain MPO activity after EM treatment for 14 months must be better addressed in future studies.

Neutrophils produce protein and lipid signals that are needed in inflammation and play an important role in regulating adaptive immune responses, thus acting in the resolution and regeneration that follows inflammation (38). In other words, neutrophils generate anti-inflammatory, pro-regenerative agents, such as resolvins, protectins, and fibroblast growth factors (39). In addition, ω-3 and its metabolites can modulate ROS production by neutrophils since neutrophils incubated with DHA improved their phagocytic and fungicidal activities (40).

The present study had some limitations since the mice were neither exposed to environmental challenges nor transgenic for neurodegenerative diseases. However, previous studies reveal that the benefits of omega oil treatment seem higher in organisms exposed to hits. Therefore, it is important to conduct further studies under exposure to adverse conditions.

In the present study, we observed that the administration of EM with a mixture of 3% ω-6:ω-3 (1.4:1.0) and ω-9:ω-6 (3.7:1.0) oils in mice from infancy to middle age caused antidepressant-like effects in mice treated for 14 months. Additionally, we observed that mice treated for 10 months presented anxiolytic-like alterations. These behavioral alterations were accompanied by a decrease in lipid peroxidation in the hippocampus and striatum of 10-month treated mice and increased GSH levels and MPO activity in 14-month treated mice. Finally, EM was found to be a promising nutraceutical option for preventing aging-related oxidative alterations in the aging animal model. Despite this, the increased immobility time observed in 10-month EM-treated mice and the higher MPO activity in 14-month EM-treated mice must be better addressed in future studies to understand the real benefits of this antioxidant strategy. Therefore, future research must determine which daily doses of the oil mix confer neuroprotective properties and elucidate the mechanisms involved in this process in genetically modified mice with increased risk for age-dependent development of neurodegenerative disorders not only in control mice as in the present study. Thus, the study showed new evidence of the controversial effects of ω-3, ω-6, and ω-9 oils, opening new perspectives for studies about their actions associated with aging.
